# Elevated levels of the angiogenic cytokines basic fibroblast growth factor and vascular endothelial growth factor in sera of cancer patients.

**DOI:** 10.1038/bjc.1997.368

**Published:** 1997

**Authors:** L. Y. Dirix, P. B. Vermeulen, A. Pawinski, A. ProvÃ©, I. Benoy, C. De Pooter, M. Martin, A. T. Van Oosterom

**Affiliations:** Department of Oncology, Catholic University of Leuven, Gasthuisberg, Belgium.

## Abstract

The concentration of basic fibroblast growth factor (bFGF) and vascular endothelial growth factor (VEGF) was determined in the serum of 90 untreated and 42 treated metastatic cancer patients, including patients with colorectal, breast, ovarian and renal carcinomas, with an enzyme-linked immunosorbent assay (ELISA). Levels higher than the 95th percentile of the concentrations of a control group, i.e. 7.5 pg ml(-1) for bFGF and 500 pg ml(-1) for VEGF, were identified as 'elevated'. One measurement during follow-up was included into the analysis per patient. For 19 treated patients, consecutive serum samples were analysed. Fifty-seven per cent of all untreated patients had elevated serum levels of one or both angiogenic factors. The fraction of patients with elevated serum levels of bFGF and/or VEGF was similar in the different tumour types. Agreement of bFGF levels and VEGF levels, classified in relation to their respective cut-off values, was present in 67% of all patients. Fifty-eight per cent of the patients with progressive disease during treatment compared with 15% of the patients showing response to treatment (chi-squared test P < 0.05) had elevated bFGF and/or VEGF serum levels. When consecutive serum samples were analysed, two-thirds of the patients showing progressive disease had increasing serum levels of the angiogenic factors compared with less than one-tenth of the patients showing response (chi-squared test P < 0.05). The lack of association between the serum bFGF and VEGF levels and the tumour type may suggest an aspecific host reaction responsible for solid tumour-related angiogenesis. The main determinants of the serum bFGF and VEGF concentration are the progression kinetics of the metastatic carcinomas.


British Journal of Cancer (1997) 76(2), 238–243
© 1997 Cancer Research Campaign

### Elevated levels of the angiogenic cytokines basic fibroblast growth factor and vascular endothelial growth factor in sera of cancer patients

LY Dirix[1,2], PB Vermeulen[1], A Pawinski[1], A Prové[3], I Benoy[3], C De Pooter[2,3], M Martin[1,4] and AT Van Oosterom[1]

Angiogenesis Group, Departments of Oncology, [1]Catholic University of Leuven, Gasthuisberg, Leuven; [2]St Augustinus Hospital, Antwerpen; [3]University of Antwerp, Universiteitsplein 1, Antwerpen; [4]Clinical Biology, University Hospital Antwerp, Antwerpen, Belgium

**Summary** The concentration of basic fibroblast growth factor (bFGF) and vascular endothelial growth factor (VEGF) was determined in the serum of 90 untreated and 42 treated metastatic cancer patients, including patients with colorectal, breast, ovarian and renal carcinomas, with an enzyme-linked immunosorbent assay (ELISA). Levels higher than the 95th percentile of the concentrations of a control group, i.e. 7.5 pg $ml^{-1}$ for bFGF and 500 pg $ml^{-1}$ for VEGF, were identified as 'elevated'. One measurement during follow-up was included into the analysis per patient. For 19 treated patients, consecutive serum samples were analysed. Fifty-seven per cent of all untreated patients had elevated serum levels of one or both angiogenic factors. The fraction of patients with elevated serum levels of bFGF and/or VEGF was similar in the different tumour types. Agreement of bFGF levels and VEGF levels, classified in relation to their respective cut-off values, was present in 67% of all patients. Fifty-eight per cent of the patients with progressive disease during treatment compared with 15% of the patients showing response to treatment (chi-squared test $P < 0.05$) had elevated bFGF and/or VEGF serum levels. When consecutive serum samples were analysed, two-thirds of the patients showing progressive disease had increasing serum levels of the angiogenic factors compared with less than one-tenth of the patients showing response (chi-squared test $P < 0.05$). The lack of association between the serum bFGF and VEGF levels and the tumour type may suggest an aspecific host reaction responsible for solid tumour-related angiogenesis. The main determinants of the serum bFGF and VEGF concentration are the progression kinetics of the metastatic carcinomas.

**Keywords:** angiogenesis; solid tumours; basic fibroblast growth factor; vascular endothelial cell growth factor; enzyme-linked immunosorbent assay

Urine and other body fluids of cancer patients have been shown to contain endothelial chemokinetic and proliferative activity (Chodak et al, 1981, 1988; Li et al, 1994). After the development of sensitive enzyme immunoassays for bFGF (Watanabe et al, 1991) and VEGF (Kondo et al, 1994), elevated levels of both angiogenic factors have been detected in the serum and the urine of patients with various types of cancer (Nguyen et al, 1994; Yamamoto et al, 1996) and have been detected significantly less frequently in individuals with no sign of cancer, ongoing inflammation or wound healing. This suggests that the induction of angiogenesis in human cancer is at least partly mediated by bFGF and VEGF.

New vessel development is required for tumour cell proliferation, extracellular matrix invasion and haematogenous metastasis (Folkman, 1990). The extent of the vasculature assessed by immunohistochemistry and microvessel counting predicts prognosis in many tumour types (Weidner et al, 1996).

bFGF and VEGF are two potent heparin-binding mediators of angiogenesis with a synergistic effect in vitro (Goto et al, 1993) and in vivo (Asahara et al, 1995). Both angiogenic factors are involved in an autocrine endothelial cell mitogenic loop (Schweigerer et al, 1987; Liu et al, 1995). VEGF receptors are expressed only on endothelial cells (Keck et al, 1989). Low oxygen tension induces the expression of bFGF and VEGF in host-derived cells and in tumour cells (Shweiki et al, 1992; Kuwabara et al, 1995).

There is a lack of quantitative data comparing the serum concentration of bFGF and VEGF in cancer patients. We initiated a study using two commercially available ELISA kits to investigate the association of serum bFGF and VEGF levels with clinical parameters of disease progression kinetics in untreated and treated metastatic breast, colorectal, ovarian and renal adenocarcinoma patients. Our results are discussed and compared with previously published data on serum bFGF and VEGF measurements.

### MATERIALS AND METHODS

From January 1995 on, serum samples were taken from 146 patients. In 14 operable colon cancer patients, samples were collected before and one month after surgery and from 132 patients who had developed distant metastasis during follow-up. Out of these 132 patients, 42 patients received some form of chemo- or hormonotherapy during the follow-up period. In 16 of these treated metastatic patients, a colorectal adenocarcinoma had been diagnosed, in 17 a breast adenocarcinoma, in six an ovarian and in three a renal adenocarcinoma. Of the 90 untreated patients, 46 had a colorectal adenocarcinoma, 22 a breast adenocarcinoma, 10 an ovarian and 12 a renal carcinoma. Ten millilitres of venous blood was drawn into a serum separator tube (type vacutainer code 607213 Becton-Dickinson) and immediately

*Received 30 September 1996*
*Revised 8 January 1997*
*Accepted 16 January 1997*

*Correspondence to:* LY Dirix, Department of Oncology, University Hospital Antwerp, Wilrijkstraat 10, B-2650 Edegem, Belgium

**Figure 1** Correlation of serum bFGF and VEGF levels of 121 patients. Each dot represents the bFGF and VEGF serum level of one patient (simple linear regression: $r = 0.21$; $P = 0.02$). All samples were obtained before the initiation of any form of therapy centrifuged at 3000 r.p.m. for 10 min. Thereafter the serum was separated and aliquoted in 1.0-ml fractions and stored at –80°C. Patient follow-up information was entered into a computerized database.

To analyse the effect of tumour progression on the bFGF and VEGF serum levels, the progression kinetics were determined in 89 out of the 132 patients with metastatic disease, based on radiological documents or, for ovarian cancer, on CA125 serum levels, both taken at least within a 3-month interval. Rapid progression was defined as a doubling within 6 months or less of the product of the largest perpendicular diameters of any measurable metastatic lesion, measured either on plain film or computerized tomography images, or as a doubling of the CA125 serum level within 3 months or less. Patients receiving treatment and having a response or a stable disease were excluded from this part of the analysis. Nineteen of the 89 patients progressed under treatment.

For the analysis of the effect of treatment on bFGF and VEGF serum levels, World Health Organization (WHO) criteria for response, no change and progressive disease were used. Tumour response was defined as a decrease in the product of the largest perpendicular tumour diameters or in the CA125 serum level of at least 50%. Progressive disease was present if the values of the parameters had increased by 25% or more. All other situations were categorized as a no change.

Successive serum levels of bFGF and VEGF were determined during the follow-up period (mean duration of 11.3 months; median 11.8 months) using two ELISA kits of R&D Systems, Minneapolis, MN, USA (Quantikine High Sensitivity human FGF basic and Quantikine human VEGF). Within-assay reproducibility was evaluated on duplicates in a subset of 115 samples. Elevated bFGF and VEGF levels were defined as being greater than the 95th percentile value in the normal control subject group described by R&D Systems. This resulted in a cut-off value for bFGF of 7.5 pg ml$^{-1}$ and for VEGF of 500 pg ml$^{-1}$. When classified in relation to the cut-off value, accordance was present in 85% of the patients for successive bFGF serum levels during the follow-up period. The same accordance rate was obtained for VEGF. The value of the first measurement done during the follow-up period was therefore taken for further analysis. Such a bFGF serum concentration value was available for 123 patients. A VEGF serum concentration value was available for 129 patients. In 121 patients, serum levels of both angiogenic factors were available.

**Figure 2** bFGF ($n = 13$) and VEGF ($n = 14$) pre- and post-operative serum levels in colorectal cancer patients

In 12 treated colorectal and seven treated breast adenocarcinoma patients, successive serum concentrations were used for further analysis.

Statistical analysis was performed with the Statview 4.0 statistical software application (Abacus Concepts) on an Apple Macintosh personal computer. The correlation between bFGF and VEGF serum concentration in the same sample was analysed by a simple linear regression model. Comparison of pre- and post-operative levels of bFGF and VEGF was performed using a Wilcoxon ranking test. The difference in the fraction of patients with elevated serum bFGF or VEGF levels in the groups investigated was analysed by a Chi-squared test. Mean bFGF and VEGF serum levels were compared using a Mann–Whitney $U$-test. A $P$-value $< 0.05$ was considered to be significant.

### RESULTS

When VEGF serum levels were determined in duplicate, 96% (110 of 115 samples) of the results had a coefficient of variation of less than 10%, and 61% (70 of 115 samples) had a coefficient of variation of less than 5%. For bFGF, the respective fractions were 73% (78 of 107 samples) and 41% (44 of 107 samples). Agreement of bFGF and VEGF serum levels when classified according to the respective cut-off values per patient was present in 67% (81 of 121 patients) of the patients. A significant, though weak, correlation was found between absolute bFGF and VEGF serum levels ($n = 121$; $r = 0.21$; $P < 0.05$) (Figure 1).

© Cancer Research Campaign 1997

**Figure 3** Comparison of VEGF ($n$ = 88) and bFGF ($n$ = 83) serum levels according to the tumour type in the group of untreated metastatic tumour patients. Mean and standard deviation are shown by the bars. In the bars the mean ± standard deviation(median) serum level is given for each tumour. The fractions of patients with serum levels of bFGF, VEGF and of VEGF and/or bFGF above the respective cut-off value for the different tumour types are given in the boxes: number of patients with elevated serum level(s)/total number (percentage). No differences were present between the tumour types ($P$ > 0.05: Mann–Whitney $U$-test and chi-squared test)

**Figure 4** Comparison of VEGF and bFGF serum levels according to response to chemo-, hormonotherapy ($n$ = 41; 15 colorectal, 18 breast, six ovarian and two renal cancer patients). Each dot represents the serum level of one patient. The values indicate the fraction of patients with a VEGF or bFGF serum level above the cut-off of 500 pg ml$^{-1}$ and 7.5 pg ml$^{-1}$ respectively. The boxes above the graphs indicate the mean ± standard deviation(median) of the serum levels (pg ml$^{-1}$) of the respective patient groups. *indicates a $P$-value of 0.025 and ** a $P$-value of 0.004 related to the differences of the mean values analysed by Mann–Whitney $U$-test. ***indicates a $P$-value of 0.025 of the difference in distribution between both groups marked, analysed with Fisher's exact chi-squared test

The majority of the patients with elevated preoperative bFGF or VEGF serum levels had normalized levels 1 month after surgery (Figure 2). Mean preoperative bFGF levels were 11.4 pg ml$^{-1}$ (standard deviation 10.6; median 8.8; $n$ = 13). Post-operative bFGF levels were significantly lower (Wilcoxon ranking test $P < 0.05$) (mean 6.4 pg ml$^{-1}$; standard deviation 6.4; median 4.3). Mean preoperative VEGF levels were 479 pg ml$^{-1}$ (standard deviation 461; median 325; $n$ = 14). Post-operative VEGF levels were not significantly lower (Wilcoxon ranking test $P$ = 0.1) (mean 311 pg ml$^{-1}$; standard deviation 233; median 223). Only one patient had a normal preoperative serum level (VEGF), which was elevated after surgery. The patient with an elevated VEGF serum level (666 pg ml$^{-1}$) before surgery and a higher still level (965 pg ml$^{-1}$) after surgery had developed a gastric ulcer in the period between the two measurements.

When all the untreated metastatic cancer patients were analysed, 57% (46 of 81 patients) had an elevated serum level of at least one of the angiogenic factors. For bFGF, 40% (33 of 83 patients) and for VEGF 40% (35 of 88 patients) had an elevated serum level. bFGF serum levels ranged from 0.0 pg ml$^{-1}$ to 33.0 pg ml$^{-1}$ ($n$ = 83;

 © Cancer Research Campaign 1997

mean 9.0; standard deviation 9.7; median 5.1). VEGF serum levels ranged from 40 pg $ml^{-1}$ to 1650 pg $ml^{-1}$ ($n$ = 88; mean 452; standard deviation 322; median 367 pg $ml^{-1}$). There was no difference in the fraction of patients with elevated serum levels, nor in the mean serum levels, when the tumour types were compared (Figure 3). The difference in the fraction of breast cancer patients with an elevated bFGF serum level (58%) and colorectal cancer patients with an elevated bFGF serum level (33%) showed a trend towards significance (chi-squared test $P = 0.06$).

Fifty-five per cent of the patients (49 of 89 patients) for whom the tumour progression rate could be estimated were defined as having a rapid progression of disease. The other 40 patients were categorized as the slowly progressive group. Of the patients with rapid progression, 93% (40 of 43 patients) had elevated serum levels of bFGF and/or VEGF compared with only 13% (5 of 38 patients) in the other group (Chi-squared test $P < 0.0001$). The corresponding fractions for bFGF alone were 72% vs 3% and for VEGF alone were 63% vs 15% (Chi-squared test $P < 0.0001$). The mean bFGF serum level in the fast-progression group was 14.7 pg $ml^{-1}$ (standard deviation 10.2; range 0.3–33.0; median 10.7 pg $ml^{-1}$) vs 2.9 pg $ml^{-1}$ in the slow-progression group (standard deviation 2.1; range 0.1–9.4; median 2.6 pg $ml^{-1}$) (Mann–Whitney $U$-test $P < 0.0001$). The mean VEGF serum level in the fast-progression group was 619 pg $ml^{-1}$ (standard deviation 381; range 40–1650; median 597 pg $ml^{-1}$) vs 324 pg $ml^{-1}$ in the slow-progression group (standard deviation 195; range 70–999; median 289 pg $ml^{-1}$) (Mann–Whitney $U$-test $P < 0.0001$).

Patients receiving chemo- or hormonotherapy and having a progressive disease had higher bFGF serum levels than the patients showing tumour response, and a larger fraction of the former group had elevated bFGF and/or VEGF serum levels (Figure 4 and Table 1).

When consecutive serum samples were analysed in the 19 treated patients, 63% (5 of 8 patients) with progressive disease had increasing levels during follow-up of at least one of the angiogenic factors compared with 9% (1 of 11 patients) in the responding patients (continuity corrected chi-squared test $P < 0.05$). Decreasing levels were found in 0% (0 of 8 patients) of the progressive disease group compared with 55% (6 of 11 patients) of the responding patients (chi-squared test, Fisher's Exact $P < 0.05$).

### DISCUSSION

We present quantitative data on the concentration of serum bFGF and VEGF in 132 patients with metastatic tumours of various histologies and in 14 patients with operable colorectal carcinoma, using an ELISA method. Elevated preoperative angiogenic factor serum levels were normalized after surgery in most of the patients. Our main observations in the patients with advanced carcinomas were the lack of correlation of bFGF and VEGF serum levels with the tumour type and the strong correlation of serum levels of both angiogenic factors with tumour progression kinetics.

Four out of ten untreated metastatic cancer patients had an elevated bFGF serum level and the same fraction had an elevated VEGF serum level. About 60% of all untreated patients had elevated serum levels of one or both angiogenic factors. This fraction was raised to more than 90% when only patients with rapid progressive disease, including patients receiving treatment, were considered. Mean bFGF serum levels were five times higher in the fast-progression patient group than in the slow-progression group. A twofold difference in favour of the former group was observed for VEGF serum levels.

Removal of a primary colorectal adenocarcinoma clearly decreased the individual serum levels of bFGF and VEGF. This suggests that the source of both angiogenic factors is present in the tumour tissue. The same observation has been made for VEGF by Yamamoto et al (1996) and for bFGF by Sliutz et al (1995) in primary breast cancer.

Nguyen et al (1994) have measured bFGF levels in the urine of patients with different types of solid tumours. Elevated bFGF was defined as a urinary level greater than the 90th percentile level of normal control subjects. The fraction of patients with elevated urinary bFGF was 45% in the metastatic patient group compared with 31% in the group of patients with local tumour growth. The proportions of patients with elevated urinary bFGF levels were similar in the different histology groups. Yamamoto et al (1996) have compared serum VEGF levels in 241 patients with mainly primary or recurrent breast and gastric cancer using an enzymatic immunoassay. The cut-off level was calculated as the 95th percentile of the level of normal controls. The rate of elevation of the VEGF serum levels was not related to the tumour type and averaged 15%. When recurrent breast cancer patients were analysed separately, 29% had high circulating VEGF levels, a fraction similar to our result of 38% in the metastatic breast cancer patient group. Toi et al (1996) measured intratumoral protein levels of VEGF in 135 primary breast cancers. They observed a wide variation in VEGF content and a significant relationship between the tumour VEGF concentration and the microvessel density as determined by immunohistochemical vessel counting. Berger et al (1995) studied the expression of VEGF mRNA in 65 human tumour xenografts of various histologies in athymic nude mice. The major disadvantage of the model is that only human tumour cell-derived VEGF mRNA was measured, and thus the host stromal response was neglected. High expression was defined as being at least 20% of the expression level of a positive control prostate cancer. High expression levels were observed in 34% of all xenografts. VEGF mRNA content of the xenografts was similar in all tumour types with the exception of renal cell carcinomas. Seven out of a total of 10 renal cell carcinoma xenografts exhibited a high VEGF mRNA expression. In our study, metastatic renal cancer patients had the highest absolute serum bFGF and VEGF levels. The fraction of renal cancer patients with at least one or both angiogenic factor serum levels elevated was also up to 14% higher than in the cancer patients with other tumours. These differences were not significant.

**Table 1** Comparison of the fraction of patients with bFGF and/or VEGF serum levels above the respective cut-off levels according to response to chemotherapy ($n$ = 41: 15 colorectal, 18 breast, six ovarian and two renal cancer patients)

| | bFGF and/or VEGF serum levels above cut-off | | |
|---|---|---|---|
| Progression | 11/19 (58)[a] | $P = 0.070$ (Progression vs No change) | $P = 0.013$ (Progression vs Response) |
| No change | 2/9 (22) | | |
| Response | 2/13 (15) | | |

[a]Number of patients with elevated level(s) / total number (percentage). $P$-values are related to chi-squared tests.

© Cancer Research Campaign 1997

The lack of difference in bFGF and VEGF levels measured in body fluids and tumour tissue between patients with various tumour types might indicate that host characteristics prevail on the tumour cell phenotype in determining the extent of angiogenesis. A discrepancy between the amount of bFGF produced by a colorectal adenocarcinoma cell line in vitro and by xenografted tumours derived from the same cell line has recently been reported (McCarty et al, 1995). The increase in bFGF levels in vivo resulted primarily from the presence of 40% of host cells within the xenografted neoplasms. Different stromal cell types have been found to express angiogenesis-modulating factors in different tumour types (reviewed in Leek et al, 1994).

The positive association of the high progression rate of the metastatic lesions with the circulating levels of bFGF and VEGF indirectly supports the hypothesis stated by Folkman et al (1990) that tumour growth is dependent on angiogenesis. bFGF and VEGF are potent and synergistically acting cytokines with proven angiogenic activity both in vitro and in vivo (Goto et al, 1993; Asahara et al, 1995). The tumour cell proliferation fraction is one of the factors contributing to tumour volume increase and has been related to bFGF and VEGF expression in several studies. In histological sections of head and neck squamous cell carcinomas, the tumour cell bromodeoxyuridine labelling index was five times higher in the bFGF-positive areas than in the bFGF-negative areas (Schultz-Hector et al, 1993). Endothelial cell labelling indices were associated with regional differences in bFGF expression in the same way as in a murine squamous cell carcinoma model. Endothelial cell doubling times were about 2.5 times shorter in the bFGF-positive tumour regions. In a series of 52 colon adenocarcinomas, the intensity of VEGF immunostaining, but not of bFGF immunostaining, correlated with the fraction of proliferating cell nuclear antigen (PCNA)-positive tumour cell nuclei in the sections (Takahashi et al, 1995). The intensity of staining of VEGF and bFGF was graded on a scale from 0 to 3. Proliferation was evaluated by counting the fraction of PCNA-positive staining tumour cells at the invasive edge. Also, in a series of 91 epidermoid lung carcinomas, a close correlation of VEGF expression with tumour cell proliferation was found (Mattern et al, 1996). In colorectal adenocarcinomas, Vermeulen et al (1995) have reported an intratumoral topographical correlation between microvessel density, Ki67 endothelial cell labelling index and Ki67 tumour cell labelling index. The finding of a positive association of microvessel density and tumour cell proliferation was supported in another study on colon cancer (Takahashi et al, 1995) but not in breast cancer (Fox et al, 1993) nor in lung cancer (Mattern et al, 1996).

We have recently reported that the positive association of a short tumour volume-doubling time with elevated bFGF and VEGF serum levels in advanced colorectal cancer is largely independent of the metastatic pattern and the extent of the disease (Dirix et al, 1996). The observation that most of the patients with progressive disease during treatment have increasing angiogenic factor serum levels might indicate that there is a minor effect of tumour mass.

Data for sarcomas and lymphomas, two tumour types for which grade reliably predicts the course of clinical progression, confirm the results of this study. Patients with high-grade malignancies, being characterized by fast progression, were found to have about five times higher bFGF and VEGF serum levels than the patients with low-grade sarcomas or lymphomas (Dirix et al, 1996).

The level of bFGF and VEGF expression had an impact on the survival of the cancer patients included in the studies of Nguyen et al (1994) and Berger et al (1995). Survival among cancer patients at the median follow-up time was 85% for individuals with a normal urinary bFGF concentration compared with 71% for individuals with an elevated bFGF level (Kaplan–Meier analysis, $P = 0.0007$). In the study of Berger et al (1995) measuring mRNA expression of VEGF in human tumour xenografts obtained from 65 patients with different histologies, the overall 5-year survival was about 20% lower in the high-VEGF expression group compared with the low-VEGF expression group. Serum VEGF concentration in 137 breast cancer patients was associated with microvessel density (Yamamoto et al, 1996). In the patients with less than 100 microvessels per mm$^2$, only 2.9% had an elevated serum VEGF. In those with more than 150 microvessels per mm$^2$, 42.9% had an elevated serum VEGF. Indirectly, VEGF serum levels might thus be related with prognosis as high microvessel density has been associated with shortened survival in many studies (Gasparini et al, 1995).

The positive association of bFGF and VEGF expression with tumour and endothelial cell proliferation, with tumour growth kinetics in treated and untreated patients and with early relapse warrants a prospective controlled study to investigate the value of the angiogenic profile of an individual cancer patient to predict outcome and response to therapy. More than half of the metastatic patients and almost all patients showing fast progression had high circulating levels of at least one of the two angiogenic factors studied. The design of an anti-angiogenic treatment directed at blocking the action of bFGF or VEGF might therefore be a reasonable therapeutic approach. Given the high degree of agreement (67%) between elevated bFGF and VEGF levels in the metastatic patient group, a combined anti-bFGF and anti-VEGF treatment might be necessary. Moreover, the redundancy will probably not be restricted to bFGF and VEGF. Hepatocyte growth factor serum levels were also found to be above the cut-off value in 37% of 134 patients with a primary breast tumour (Taniguchi et al, 1995). A therapeutical strategy that will inhibit more advanced steps in the process of new vessel development might not have to deal with this biological redundancy.

### ACKNOWLEDGEMENTS

Peter Vermeulen is the holder of a grant from 'Vlaamse Kankerliga'. The technical support of Ilse Vanhoolst was greatly appreciated.

### REFERENCES

Asahara T, Bauters C, Zheng LP, Takeshita S, Bunting S, Ferrara N, Symes JF and Isner JM (1995) Synergistic effect of vascular endothelial growth factor and basic fibroblast factor on angiogenesis in vivo. *Circulation* **92**: 365–371

Berger DP, Herbstritt L, Dengler WA, Marme D, Mertelsmann R and Fiebig HH (1995) Vascular endothelial growth factor (VEGF) mRNA expression in human tumor models of different histologies. *Ann Oncol* **6**: 817–825

Chodak GW, Scheiner CJ and Zetter BR (1981) Urine from patients with transitional cell carcinoma stimulates migration of capillary endothelial cells. *N Engl J Med* **305**: 869–874

Chodak GW, Hospelhorn V, Judge SM, Mayforth R, Koeppen H and Sasse J (1988) Increased levels of fibroblast growth factor-like activity in urine from patients with bladder or kidney cancer. *Cancer Res* **48**: 2083–2088

Dirix LY, Vermeulen PB, Hubens G, Benoy I, Martin M, De Pooter C and Van Oosterom (1996) Serum basic fibroblast growth factor and vascular endothelial growth factor and tumour growth kinetics in advanced colorectal cancer. *Ann Oncol* **7**: 843–848

Folkman J (1990) What is the evidence that tumors are angiogenesis dependent? *J Natl Cancer Inst* **82**: 4–6

© Cancer Research Campaign 1997

Fox SB, Gatter KC, Bicknell R, Going JJ, Stanton P, Cooke TG and Harris AL (1993) Relationship of endothelial cell proliferation to tumor vascularity in human breast cancer. *Cancer Res* **53**: 4161–4163

Gasparini G and Harris AL (1995) Clinical importance of the determination of tumor angiogenesis in breast carcinoma: much more than a new prognostic tool. *J Clin Oncol* **13**: 765–782

Goto F, Goto K, Weindel K and Folkman J (1993) Synergistic effects of vascular endothelial growth factor and basic fibroblast growth factor on the proliferation and cord formation of bovine capillary endothelial cells within collagen gels. *Lab Invest* **69**: 508–517

Kondo S, Asano M, Matsuo K, Ohmori I and Suzuki H (1994) Vascular endothelial growth factor/vascular permeability factor is detectable in the sera of tumor-bearing mice and cancer patients. *Biochim Biophys Acta* **1221**: 211–214

Kuwabara K, Ogawa S, Matsumoto M, Koga S, Clauss M, Pinsky DJ, Lyn P, Leavy J, Witte L, Joseph-Silverstein J, Furie MB, Torcia G, Cozzolino F, Kamada T and Stern DM (1995) Hypoxia-mediated induction of acidic/basic fibroblast growth factor and platelet-derived growth factor in mononuclear phagocytes stimulates growth of hypoxic endothelial cells. *Proc Natl Acad Sci* **92**: 4606–4610

Keck PJ, Hauser SD, Krivi G, Sanzo K, Warren T, Feder J and Connolly DT (1989) Vascular permeability factor, an endothelial cell mitogen related to PDGF. *Science* **246**: 1309–1312

Leek RD, Harris AL and Lewis CE (1994) Cytokine networks in solid human tumors: regulation of angiogenesis. *J Leukoc Biol* **56**: 423–435

Li VW, Folkerth RD, Watanabe H, Yu C, Rupnick M, Barnes P, Scott RM, Black PM, Sallan SE and Folkman J (1994) Microvessel count and cerebrospinal fluid basic fibroblast growth factor in children with brain tumours. *Lancet* **344**: 82–86

Liu Y, Cox SR, Morita T and Kourembanas S (1995) Hypoxia regulates vascular endothelial growth factor gene expression in endothelial cells. Identification of a 5′ enhancer. *Circ Res* **77**: 638–643

Mattern J, Koomagi R and Volm M (1996) Association of vascular endothelial growth factor with intratumoral microvessel density and tumour cell proliferation in human epidermoid lung carcinoma. *Br J Cancer* **73**: 931–934

McCarty LP, Karr SM, Harris BZ, Michelson SG and Leith JT (1995) Comparison of basic fibroblast growth factor levels in clone A human colon cancer cells in vitro with levels in xenografted tumours. *Br J Cancer* **72**: 10–16

Nguyen M, Hiroyuki W, Budson AE, Richie JP and Folkman J (1993) Elevated levels of the angiogenic peptide basic fibroblast growth factor in urine of bladder cancer patients. *J Natl Cancer Inst* **85**: 24–25

Schultz-Hector S and Haghayegh S (1993) β-fibroblast growth factor expression in human and murine squamous cell carcinomas and its relationship to regional endothelial cell proliferation. *Cancer Res* **53**: 1444–1449

Schweigerer L, Neufeld G, Friedman J, Abraham JA, Fiddes JC and Gospodarowicz D (1987) Capillary endothelial cells express basic fibroblast growth factor, a mitogen that promotes their own growth. *Nature* **325**: 257–259

Shweiki D, Itin A, Soffer D and Keshet E (1992) Vascular endothelial growth factor induced by hypoxia may mediate hypoxia-initiated angiogenesis. *Nature* **359**: 843–845

Sliutz G, Tempfer C, Obermair A, Dadak CH and Kainz CH (1995) Serum evaluation of basic FGF in breast cancer patients. *Anticancer Res* **15**: 2675–2678

Takahashi Y, Kitadai Y, Bucana CD, Cleary KR and Ellis LM (1995) Expression of vascular endothelial growth factor and its receptor, KDR, correlates with vascularity, metastasis, and proliferation of human colon cancer. *Cancer Res* **55**: 3964–3968

Taniguchi T, Toi M, Inada K, Imazawa T, Yamamoto Y and Tominaga T (1995) Serum concentrations of hepatocyte growth factor in breast cancer patients. *Clin Cancer Res* **1**: 1031–1034

Toi M, Kondo S, Suzuki H, Yamamoto Y, Inada K, Imazawa T, Taniguchi T and Tominaga T (1996) Quantitative analysis of vascular endothelial growth factor in primary breast cancer. *Cancer* **77**: 1101–1106

Vermeulen PB, Verhoeven D, Hubens G, Van Marck E, Goovaerts G, Huyghe M, De Bruijn EA, Van Oosterom AT and Dirix LY (1995) Microvessel density, endothelial cell proliferation and tumour cell proliferation in human colorectal adenocarcinomas. *Ann Oncol* **6**: 59–64

Watanabe H, Hori A, Seno M, Kozai Y, Igarashi K, Ichimori Y and Kondo K (1991) A sensitive enzyme immunoassay for human basic fibroblast growth factor. *Biochem Biophys Res Comm* **175**: 229–235

Weidner N and Folkman J (1996) Tumoral vascularity as a prognostic factor in cancer. In *Important Advances in Oncology*, DeVita VT, Hellman S and Rosenberg SA. (eds), pp. 167–190. Lippincott-Raven: Philadelphia

Yamamoto Y, Toi M, Kondo S, Matsumoto T, Suzuki H, Kitamura M, Tsuruta K, Taniguchi T, Okamoto A, Mori T, Yoshida M, Ikeda T and Tominaga T (1996) Concentrations of vascular endothelial growth factor in the sera of normal controls and cancer patients. *Clin Cancer Res* **2**: 821–826

© Cancer Research Campaign 1997


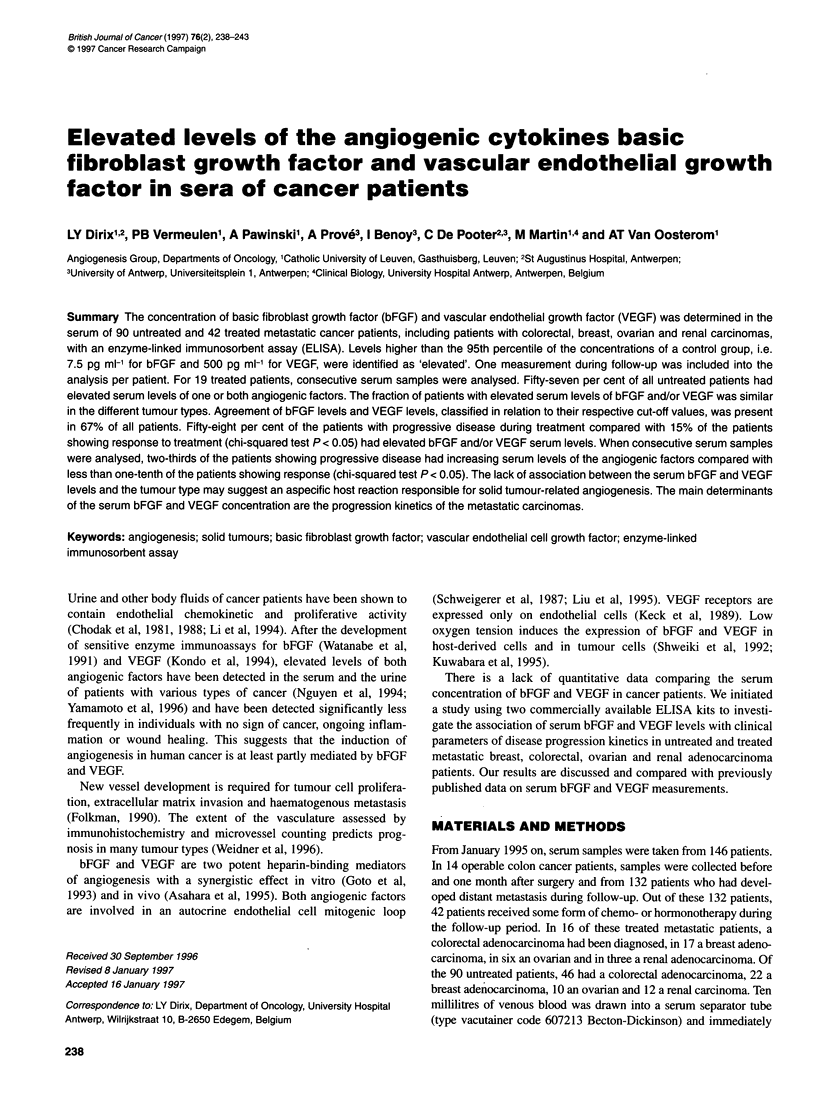

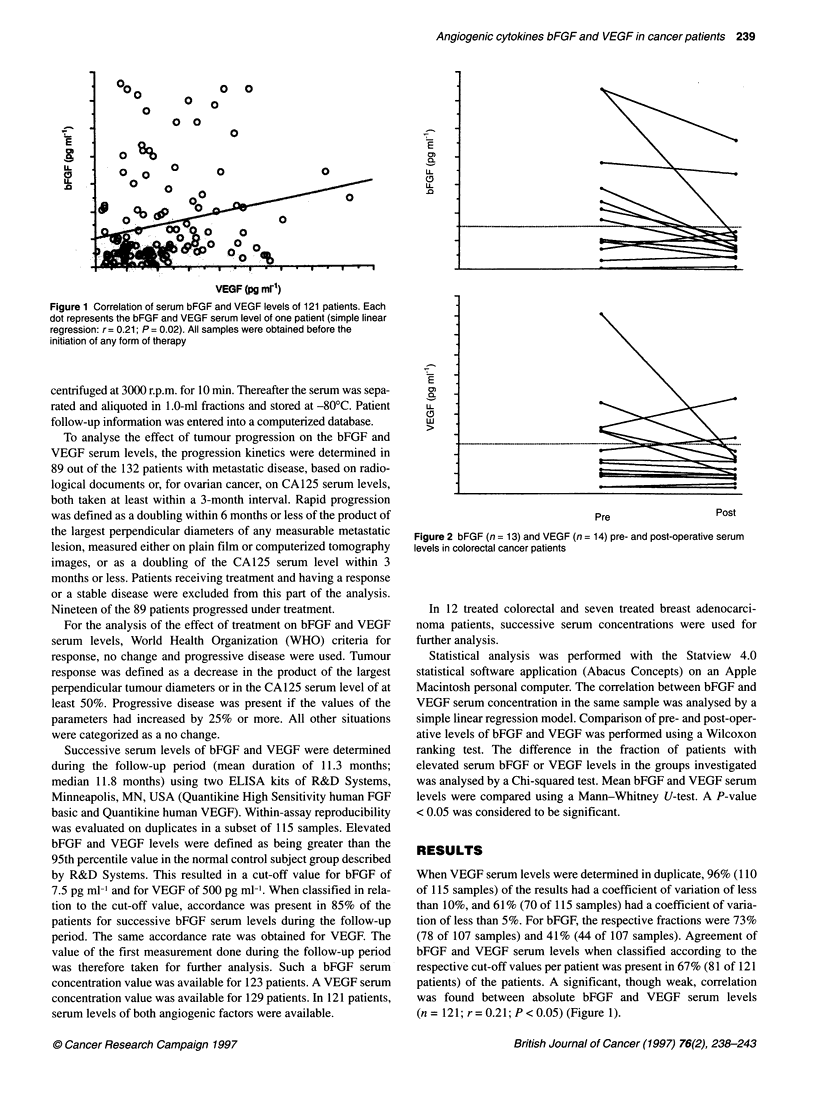

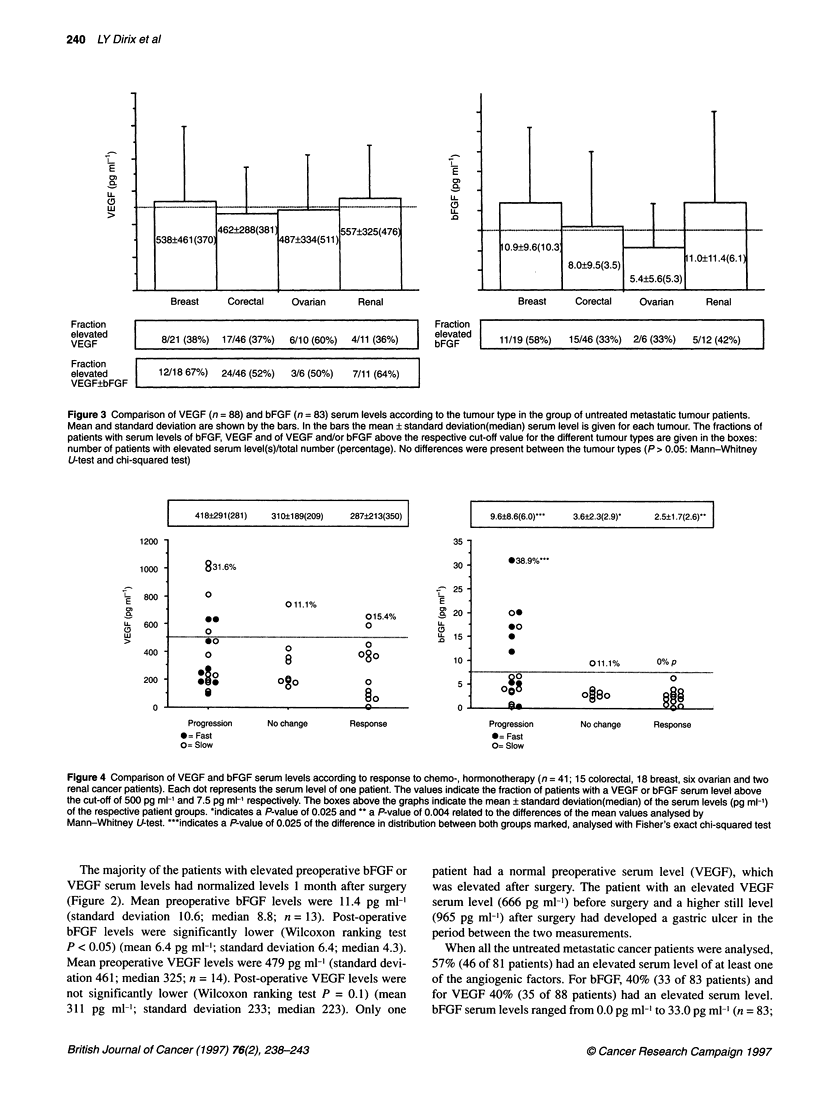

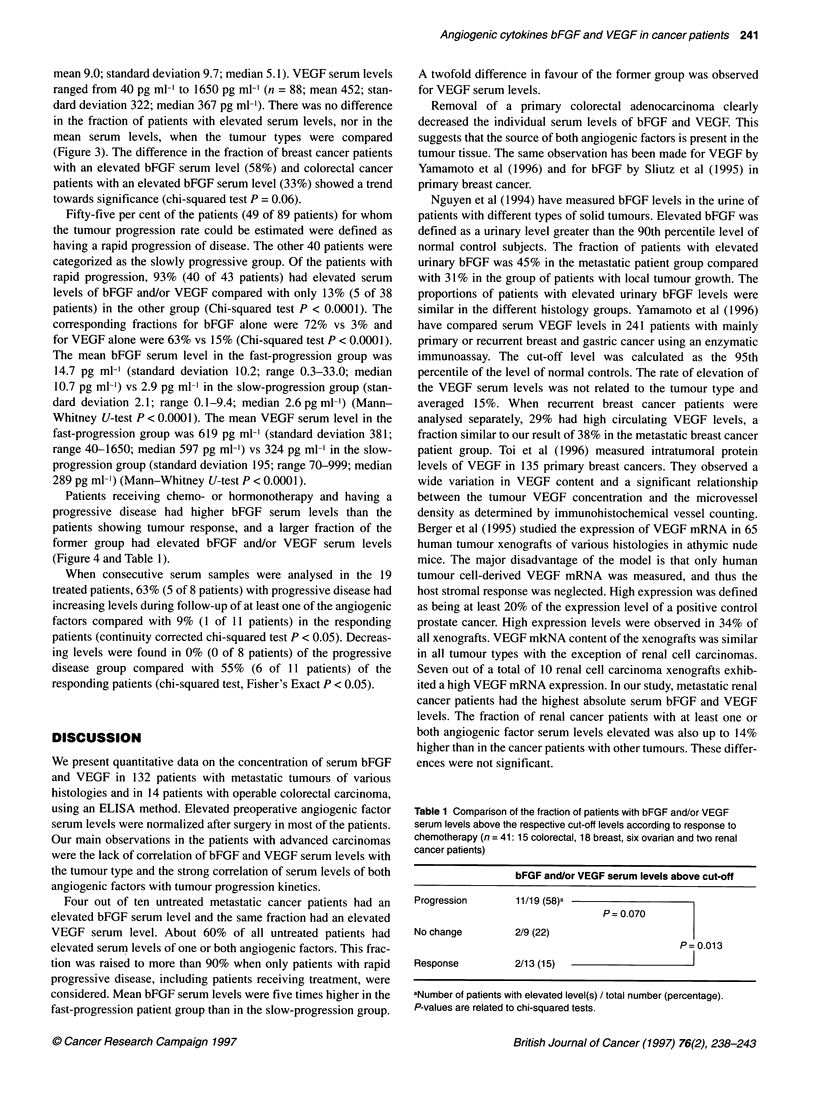

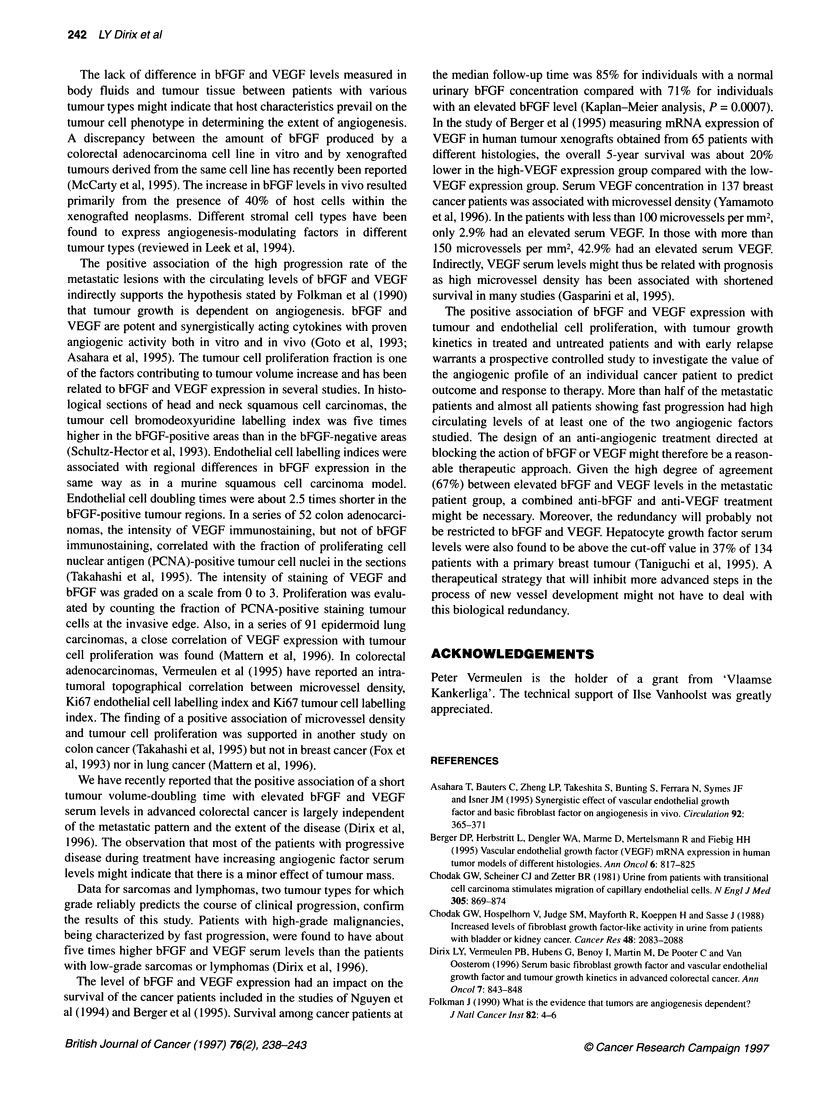

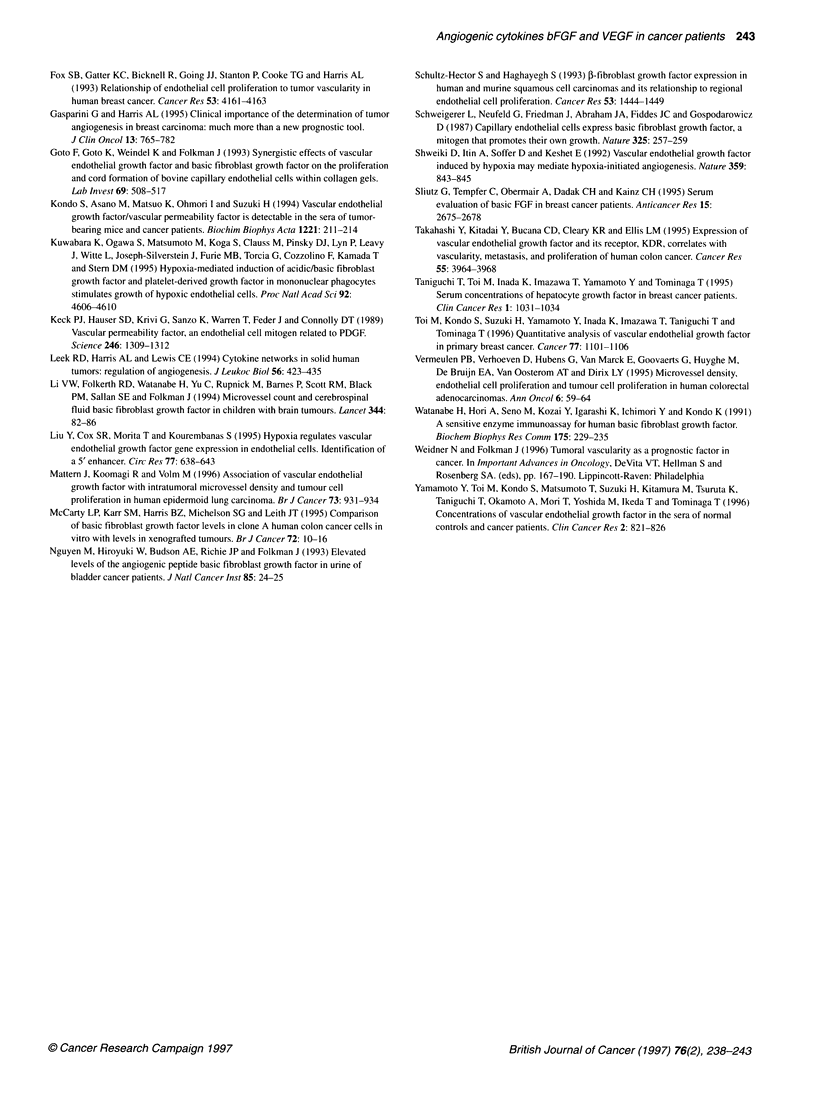


## References

[OCR_00632] Berger D. P., Herbstritt L., Dengler W. A., Marmé D., Mertelsmann R., Fiebig H. H. (1995). Vascular endothelial growth factor (VEGF) mRNA expression in human tumor models of different histologies.. Ann Oncol.

[OCR_00642] Chodak G. W., Hospelhorn V., Judge S. M., Mayforth R., Koeppen H., Sasse J. (1988). Increased levels of fibroblast growth factor-like activity in urine from patients with bladder or kidney cancer.. Cancer Res.

[OCR_00637] Chodak G. W., Scheiner C. J., Zetter B. R. (1981). Urine from patients with transitional-cell carcinoma stimulates migration of capillary endothelial cells.. N Engl J Med.

[OCR_00647] Dirix L. Y., Vermeulen P. B., Hubens G., Benoy I., Martin M., De Pooter C., Van Oosterom A. T. (1996). Serum basic fibroblast growth factor and vascular endothelial growth factor and tumour growth kinetics in advanced colorectal cancer.. Ann Oncol.

[OCR_00653] Folkman J. (1990). What is the evidence that tumors are angiogenesis dependent?. J Natl Cancer Inst.

[OCR_00661] Fox S. B., Gatter K. C., Bicknell R., Going J. J., Stanton P., Cooke T. G., Harris A. L. (1993). Relationship of endothelial cell proliferation to tumor vascularity in human breast cancer.. Cancer Res.

[OCR_00666] Gasparini G., Harris A. L. (1995). Clinical importance of the determination of tumor angiogenesis in breast carcinoma: much more than a new prognostic tool.. J Clin Oncol.

[OCR_00671] Goto F., Goto K., Weindel K., Folkman J. (1993). Synergistic effects of vascular endothelial growth factor and basic fibroblast growth factor on the proliferation and cord formation of bovine capillary endothelial cells within collagen gels.. Lab Invest.

[OCR_00690] Keck P. J., Hauser S. D., Krivi G., Sanzo K., Warren T., Feder J., Connolly D. T. (1989). Vascular permeability factor, an endothelial cell mitogen related to PDGF.. Science.

[OCR_00677] Kondo S., Asano M., Matsuo K., Ohmori I., Suzuki H. (1994). Vascular endothelial growth factor/vascular permeability factor is detectable in the sera of tumor-bearing mice and cancer patients.. Biochim Biophys Acta.

[OCR_00682] Kuwabara K., Ogawa S., Matsumoto M., Koga S., Clauss M., Pinsky D. J., Lyn P., Leavy J., Witte L., Joseph-Silverstein J. (1995). Hypoxia-mediated induction of acidic/basic fibroblast growth factor and platelet-derived growth factor in mononuclear phagocytes stimulates growth of hypoxic endothelial cells.. Proc Natl Acad Sci U S A.

[OCR_00695] Leek R. D., Harris A. L., Lewis C. E. (1994). Cytokine networks in solid human tumors: regulation of angiogenesis.. J Leukoc Biol.

[OCR_00699] Li V. W., Folkerth R. D., Watanabe H., Yu C., Rupnick M., Barnes P., Scott R. M., Black P. M., Sallan S. E., Folkman J. (1994). Microvessel count and cerebrospinal fluid basic fibroblast growth factor in children with brain tumours.. Lancet.

[OCR_00706] Liu Y., Cox S. R., Morita T., Kourembanas S. (1995). Hypoxia regulates vascular endothelial growth factor gene expression in endothelial cells. Identification of a 5' enhancer.. Circ Res.

[OCR_00711] Mattern J., Koomägi R., Volm M. (1996). Association of vascular endothelial growth factor expression with intratumoral microvessel density and tumour cell proliferation in human epidermoid lung carcinoma.. Br J Cancer.

[OCR_00716] McCarty L. P., Karr S. M., Harris B. Z., Michelson S. G., Leith J. T. (1995). Comparison of basic fibroblast growth factor levels in clone A human colon cancer cells in vitro with levels in xenografted tumours.. Br J Cancer.

[OCR_00726] Schultz-Hector S., Haghayegh S. (1993). Beta-fibroblast growth factor expression in human and murine squamous cell carcinomas and its relationship to regional endothelial cell proliferation.. Cancer Res.

[OCR_00731] Schweigerer L., Neufeld G., Friedman J., Abraham J. A., Fiddes J. C., Gospodarowicz D. (1987). Capillary endothelial cells express basic fibroblast growth factor, a mitogen that promotes their own growth.. Nature.

[OCR_00736] Shweiki D., Itin A., Soffer D., Keshet E. (1992). Vascular endothelial growth factor induced by hypoxia may mediate hypoxia-initiated angiogenesis.. Nature.

[OCR_00741] Sliutz G., Tempfer C., Obermair A., Dadak C., Kainz C. (1995). Serum evaluation of basic FGF in breast cancer patients.. Anticancer Res.

[OCR_00746] Takahashi Y., Kitadai Y., Bucana C. D., Cleary K. R., Ellis L. M. (1995). Expression of vascular endothelial growth factor and its receptor, KDR, correlates with vascularity, metastasis, and proliferation of human colon cancer.. Cancer Res.

[OCR_00753] Taniguchi T., Toi M., Inada K., Imazawa T., Yamamoto Y., Tominaga T. (1995). Serum concentrations of hepatocyte growth factor in breast cancer patients.. Clin Cancer Res.

[OCR_00758] Toi M., Kondo S., Suzuki H., Yamamoto Y., Inada K., Imazawa T., Taniguchi T., Tominaga T. (1996). Quantitative analysis of vascular endothelial growth factor in primary breast cancer.. Cancer.

[OCR_00763] Vermeulen P. B., Verhoeven D., Hubens G., Van Marck E., Goovaerts G., Huyghe M., De Bruijn E. A., Van Oosterom A. T., Dirix L. Y. (1995). Microvessel density, endothelial cell proliferation and tumour cell proliferation in human colorectal adenocarcinomas.. Ann Oncol.

[OCR_00770] Watanabe H., Hori A., Seno M., Kozai Y., Igarashi K., Ichimori Y., Kondo K. (1991). A sensitive enzyme immunoassay for human basic fibroblast growth factor.. Biochem Biophys Res Commun.

[OCR_00775] Weidner N., Folkman J. (1996). Tumoral vascularity as a prognostic factor in cancer.. Important Adv Oncol.

[OCR_00780] Yamamoto Y., Toi M., Kondo S., Matsumoto T., Suzuki H., Kitamura M., Tsuruta K., Taniguchi T., Okamoto A., Mori T. (1996). Concentrations of vascular endothelial growth factor in the sera of normal controls and cancer patients.. Clin Cancer Res.

